# Migralepsy, hemicrania epileptica, post-ictal headache and “ictal epileptic headache”: a proposal for terminology and classification revision

**DOI:** 10.1007/s10194-011-0318-4

**Published:** 2011-03-01

**Authors:** Vincenzo Belcastro, Pasquale Striano, Dorotheè G. A. Kasteleijn-Nolst Trenité, Maria Pia Villa, Pasquale Parisi

**Affiliations:** 1 Department of Neuroscience, Neurology Clinic, Sant’Anna Hospital, Como, Italy; 2 Muscular and Neurodegenerative Diseases Unit, G. Gaslini Institute, University of Genova, Genoa, Italy; 3 Department of Medical Genetics, Wilhelmina Children’s Hospital, Utrecht University Medical Centre, Utrecht, The Netherlands; 4 Child Neurology, Headache Paediatric Center, Paediatric Sleep Centre, Chair of Paediatrics, II Faculty of Medicine, “Sapienza University” c/o Sant’Andrea Hospital, Via di Grottarossa, 1035-1039 Rome, Italy

**Keywords:** Migralepsy, Hemicrania epileptica, Status migrainosus, Migraine, Status epilepticus, Epilepsy, Ictal epileptic headache, EEG

## Abstract

Despite the fact that migraine and epilepsy are among the commoner brain diseases and that comorbidity of these conditions is well known, only few reports of migralepsy and hemicrania epileptica (HE) have been published according to the current ICHD-II criteria. Particularly, ICHD-II describes “migraine-triggered seizure” (i.e., migralepsy) among complications of migraine at “1.5.5” (as a rare event in which a seizure happens during migrainous aura), while hemicrania epileptica (coded at “7.6.1”) and post-ictal headache (coded at “7.6.2”) are described among headaches attributed to epileptic seizure. However, to date neither the International Headache Society nor the International League against Epilepsy mention that headache/migraine may be the sole ictal epileptic manifestation. Based on the current knowledge, migralepsy is highly unlikely to exist as such. We, therefore, propose to delete this term until clear evidence its existence is provided. Moreover, we herein propose a revision of terminology and classification criteria to properly represent the migraine/headache relationships. We suggest the term “ictal epileptic headache” in cases in which headache/migraine is the sole ictal epileptic manifestation.

## Introduction

Migraine and epilepsy have common pathophysiologic mechanisms and share essential and defining attributes which distinguish them from other common neurological disorders: they are both characterized by paroxysmal symptoms and are, therefore, episodic disorders [1].

However, the two phenomena are sometimes difficult to be differentiated only on clinical ground. In fact, epileptic seizures and migraine attacks may be mistaken one for the other or they can even overlap [2–7]. In particular, occipital lobe seizures may be misinterpreted as migraine visual auras, even though these conditions have distinct characteristics in most cases [2, 3].

Despite almost two centuries of investigations, the relationship between epilepsy and migraine has not been completely elucidated and although there are several clinical links between both conditions only some of them have been recently incorporated into classification systems. The second edition of the International Classification of Headache Disorders (ICHD-II, 2004) [8] distinguishes three disorders, namely, migraine-triggered seizures (migralepsy), hemicrania epileptica, and postictal headaches. Noteworthy, other terms have been utilized to explain the “epilepsy-migraine” or “migraine-epilepsy” sequence, e.g., the term “intercalated seizures” has been utilized to denote epileptic seizures occurring between the migrainous aura and the headache phase of migraine [9].

Only few case reports of migralepsy [10] and hemicrania epileptica (HE) [11] have been published despite the fact that migraine and epilepsy are among the commoner brain diseases. These cases highlight the inadequacy of the current definitions of ICHD-II about the temporal and/or clinical link and overlap between migraine and epilepsy. According to the current literature data, we propose a revision for terminology and classification criteria in order to properly represent the epilepsy-migraine/headache relationships.

## Migralepsy

Migralepsy is an old term deriving from migra(ine) and (epi)lepsy that has been used for the first time by Lennox and Lennox to describe a condition in which “ophthalmic migraine with perhaps nausea and vomiting was followed by symptoms characteristic of epilepsy” [12]. After the first report, 19 additional cases have been described in the literature until 1993 when the term ‘migralepsy’ was reintroduced by Marks and Ehrenberg [13]. However, the concept of migralepsy as a migraine-epilepsy sequence has been the subject of criticism by several authors since most of the reported cases of “migralepsy” do not allow a meaningful and unequivocal migraine-epilepsy sequence and they were genuine occipital seizures imitating migraine with aura [2, 3]. In this sense, two of the three “migralepsy” patients of Lennox and Lennox seemed to have symptomatic and idiopathic occipital epilepsy with visual hallucinations [10].

Despite the scepticism surrounding the concept of migralepsy, in which two separate disorders are presumed to occur in succession, migraine-triggered seizures have been included in the recent ICHD-II (“1.5.5”) as a complication of migraine. However, migralepsy is neither included in the currently used International League Against Epilepsy (ILAE) seizure classification nor in the recent recommendations of the ILAE Commission on Classification and Terminology [14]. In particular, migralepsy, which in the recent ICHD-II is defined as a seizure developing during or within 1 h of a migraine aura, is extremely rare [10]. The distinction between migraine visual auras and occipital lobe seizures must be kept in mind when evaluating attacks suggestive of migralepsy [2, 3] and this concept has been recently reviewed by Sances and coworkers [10]. In particular, these authors underscore the high prevalence of purely epileptic disorders among cases reported as migralepsy. Indeed, among the 50 potential migralepsy cases identified in the literature only two meet the current ICHD-II criteria supporting a diagnosis of migralepsy [8]. In this regard, the case reported by Sances and colleagues [10] might open a new scenario that should not be restricted to simply the redefinition of “migralepsy concept.” Sances et al. described a patient who complained of “visual symptoms associated with a déja`-vu sensation, a smell of fresh laundry, and nausea, lasting about 10 min, which then developed into a generalized tonic clonic seizure.” Although a migrainous origin for this patient’s visual symptoms was suggested by a score of 6 in the Visual Aura Rating Scale, his clinical symptoms suggestive for a typical episode of visual aura could also represent together with the other symptoms (i.e., déja`-vu sensation, a smell of fresh laundry, and nausea) an epileptic aura that may develop into a generalized seizure. In this sense, the duration of visual symptoms plays a key role in recognizing ictal visual hallucinations suggestive of occipital lobe seizures or migraine visual aura even if the visual symptoms are not being followed by headache or an epileptic seizure [2, 3].

For above-mentioned reasons, the concept of migralepsy, according to the current definition, is too narrow and inadequate [10, 15, 16] and it should be revised keeping in the mind that headache or visual symptoms can be the epileptic “aura” of a seizure, as it has been shown in the case description of a patient with a partial status epilepticus in occipital lobe epilepsy [17].

Thus, the sequence “migraine-epilepsy” defined as “migralepsy” could often be “simply” a seizure starting with an ictal epileptic headache (IEH) [4–6] followed by a sensory-motor partial or generalized seizure. On the other hand, evidence exists that headache per se may represent an epileptic seizure and that in some individuals it may be the sole manifestation of epilepsy [6, 7, 11, 18–20].

## Hemicrania epileptica

Hemicrania epileptica is recognized as an ipsilateral headache with migrainous features occurring as an ictal manifestation of the seizure discharge [11]. In the first report, Isler and colleagues [11] found that hemicranial attacks of pain coincided with seizures activity and lasted for seconds to minutes (i.e. hemicrania epileptica). However, two exceptions were noted: (1) a case of complex status in which headache lasted for hours and, (2) a case in which the headache lasted most of the 20 min of a recorded seizure [11].

This condition, albeit rare, has been included in the recent ICHD-II [8], based on fulfilment of these criteria: (1) headache lasting seconds to minutes, with features of migraine, fulfilling criteria C and D; (2) the patient is having a partial epileptic seizure; (3) headache develops synchronously with the seizure and it is ipsilateral to the ictal discharge; (4) headache resolves immediately after the seizure. Diagnosis requires the simultaneous onset of headache with electroencephalographically (EEG)-demonstrated discharge [11].

Starting from 1988 (when the first edition of ICHD appeared) to date, we identified in the literature a total of five potential HE patients, and we reviewed them systematically to ascertain their diagnostic plausibility.

In all these patients, migraine/headache lasted longer than “seconds to minutes” and it appeared to be the sole manifestation of a non-convulsive status epilepticus (SE) [4–7, 16–20]. Notably, overall these patients did not meet current ICHD-II criteria for HE (Table 1).

**Table 1:** Review of the five reported “ictal epileptic headache” cases described in the literature compared to the present ICHD-II criteria for “Hemicrania Epileptica” (HE)

ICHD-II criteria for HE	Walker et al. [17]	Ghofrani et al. [18]	Parisi et al. [4]	Perucca et al. [19]	Belcastro et al. [20]
(a) Headache lasting seconds to minutes, with features of migraine	>72 h	NA	>72 h	>72 h	>72 h
(b) The patient is having a partial epileptic seizure	NCSE	NCSE	NCSE	NCSE	NCSE
(c) Headache develops ipsilaterally to the ictal discharge	YES	NO	YES	YES	YES
(d) Headache resolves immediately after the seizure	YES	YES	YES	YES	YES

In all cases headache represented the sole ictal manifestation and a complete remission of headache and epileptic abnormalities was obtained after anticonvulsant treatment

*HE* hemicrania epileptica, *NCSE* non-convulsive status epilepticus, *NA* not available

According to the EEG patterns, four patients showed partial status epilepticus in the occipital lobes [4–7, 16–20], while bilateral continuous spike-and slow-wave discharges (i.e. absence status) were reported by Ghofrani and colleagues [18]. These observations suggest that: (1) headache/migraine could be the sole symptom not only of a partial [4, 17, 19, 20] but also of a generalized [18] non-convulsive SE; (2) headache could be ipsi or contra-lateral to the ictal epileptiform discharge [4, 17–20].

## Post-ictal headache

Although headache with migraine features is commonly a post-ictal phenomenon, occurring in about 50% of the epileptic patients, it is often neglected because of the dramatic manifestation of the seizure. This condition has been included in the recent ICHD-II [8], based on the fulfilment of these criteria: (a) headache with features of tension-type headache or, in a patient with migraine, of migraine headache and fulfilling the criteria at points b, c and d; (b) the patient has had a partial or generalized seizure; (c) headache develops within 3 h following the seizure; (d) headache resolves within 72 h after the seizure.

Schön and Blau [21] reported on 100 epileptic patients, 51 of whom had postictal headache (PIH). In this study, PIH was more commonly associated with generalized tonic-clonic seizures than with focal seizures; 9% of the patients with PIH also had independent migraine attacks [21]. The headaches were either bilateral or unilateral. They were associated with phonophobia and photophobia, throbbing pain, vomiting, nausea, and visual aura, and lasted 6–72 h. Epileptic migraineurs recognized these headaches as being similar to their migraine [21]. PIH has been reported in patients with symptomatic epilepsy but it is mainly emphasized in idiopathic occipital seizures [22]. It may be that the seizure discharges in the occipital lobes trigger a genuine migraine headache through cortical spreading depression (CSD) and trigeminovascular or brainstem mechanisms, as previously suggested [5, 6, 23]. In this respect, as following reported, the onset of epileptic seizures may facilitate the onset of CSD to a greater degree than the onset of CSD facilitates the onset of epileptic seizures. This may explain why, in the clinical context, it is more likely to observe epileptic patients with post-ictal headache (51% according to Schön and Blau) [21] rather than migraine subjects with epileptic seizures [5, 6, 23].

## “Ictal epileptic headache”

The occipital lobe is deemed to be the brain structure most responsible for both the development of migraine [24, 25] and occipital lobe epilepsies [26]. In particular, both occipital epilepsies and migraine are characterized by visual symptoms (elementary visual hallucinations vs. aura) followed by headache and other autonomic symptoms. However, recognition of headache as an epileptic manifestation per se (rarely even the sole epileptic manifestation) still represents a challenge [2–7, 10, 11, 15–20].

So, what would be the clinical, EEG and neuroimaging features of IEH? Literature data suggest that migraine as a sole manifestation of a seizure might be the expression of a non-convulsive SE [4–7, 16–20], the nature of which could be diagnosed only by EEG recordings [6, 7, 16–20]. Unfortunately, there is no specific EEG picture but, on the contrary, different EEG patterns associated with IEH have been recorded during migraine-like complaints in these patients [6, 7, 16–20]: (1) high-voltage, rhythmic, 11–12 Hz activity with intermingled spikes over the right temporo-occipital regions [19, 20]; (2) high voltage theta activity intermingled with sharp waves over occipital region [4, 17] and, (3) bilateral continuous spike-and slow-wave discharges [18]. Furthermore, a photoparoxysmal response (PPR) [17] in combination with complaints about a light pulsating headache was seen during intermittent photic stimulation [4]. In this respect, we would also like to stress that there could even be an isolated epileptic headache without any other associated ictal epileptic manifestations nor EEG abnormalities recognizable by scalp EEG recording, whose ictal origin can be conversely demonstrated by depth electrode studies (see patient number 2 by Laplante et al. [27]); accordingly, on the other hand, as we all know, in other types of epilepsy, such as in frontal lobe epilepsy, often (from 20 to 40% of patients) it is not possible to detect any ictal epileptic activity from the scalp-EEG recording [28].

Notably, in most of these patients a complete remission of the headache and of epileptic abnormalities was obtained not with specific antimigraine drugs, but after intravenous administration of diazepam [4, 18–20] or phenytoin [19].

Regardless of the aetiology, brain MRI showed secondary brain lesions in the right temporo-parieto-occipital region with a restricted diffusion in the right occipital region [19, 20] or enlarged sulci in the right parietal region [17]. However, IEH has also been reported in patients with idiopathic epilepsy [4, 18].

## EEG contribution to study the association between migraine and epilepsy

Although EEG is not useful in the routine assessment of headache patients, there is, however, clearly a role for 24-h-closed-circuit television EEG recording. Marks and Ehrenberg [13] studied patients with migralepsy using multiple 24-h video EEG telemetry recordings. The entire migraine-epilepsy sequence of two patients was captured, showing changes during the clinical migraine aura that were atypical for electrographic epilepsy. During migraine aura, bursts of spike activity may resemble the ictal EEG during an epileptic seizure. In most reported cases, however, the EEG does not show the usual temporal evolution with progressive increase and declines in the frequency and amplitude of rhythmic, repetitive epileptiform activity typical of ictal EEGs in epilepsy [29, 30]. In addition, the EEG during migraine aura may show “waxing and waning” patterns, separated by completely normal EEG activity despite the persistence of clinical symptoms [31]. The study of EEG during paroxysmal visual manifestation (migrainous aura, epileptic seizure) would show different patterns according to the clinical symptoms (positive or deficient) [31].

As previously specified, according to our and others experiences on published IEH cases [4–7, 11, 16–20], the ictal-EEG recording during migraine-like complaints in those patients showed no specific EEG picture: (a) high-voltage, rhythmic, 11–12 Hz activity with intermingled spikes over the right temporo-occipital regions [19, 20]; (b) high voltage theta activity intermingled with sharp waves over occipital region [4, 17] and, (c) bilateral continuous spike-and slow-wave discharges [18]. In this respect, in fact, it should also be stressed that, sometimes, [27], there could even be an isolated epileptic headache without any other associated ictal epileptic manifestations nor EEG abnormalities recognizable by scalp EEG recording, whose ictal origin can be, conversely, demonstrated by depth electrode studies (see patient number 2 by Laplante et al. [27]). On the other hand, as it happens in certain types of epilepsy, such as in frontal lobe epilepsy, often (from 20 to 40% of patients) it is not possible to detect any ictal epileptic activity from the scalp-EEG recording [28].

## Mechanisms underlying “ictal epileptic headache”

A migraine/headache attack can originate at either the cortical or subcortical level, whereas an epileptic focus arises cortically and can only be modulated at the subcortical level [6]. A main mechanism has been hypothesized to explain headache/migraine as a sole “ictal epileptic manifestation” [6]: a sub-clinical epileptic discharge might activate the trigeminovascular system, resulting in a migraine/headache without any other associated cortical epileptic sign or symptom. In fact, the central autonomic networks (whether cortical or subcortical) have a lower threshold for epileptogenic activation than those that produce a focal cortical sensory-motor semiology. The threshold required for the onset of CSD has recently been suggested to be lower than that required for an epileptic seizure [5, 6, 23]. In other words, the onset of epileptic seizures may facilitate the onset of CSD to a greater degree than the onset of CSD facilitates the onset of epileptic seizures [5, 6, 23]. This may explain why, in the clinical context, it is more likely to observe epileptic patients with peri-ictal or inter-ictal migraine rather than migraine subjects with epileptic seizures [5, 6, 23].

Moreover, as regard, IEH associated with other ictal epileptic manifestations, it should be borne in mind that headache may be associated with ictal-sensory and motor features more frequently than the literature suggests. Indeed, this association might be strongly underestimated owing to impaired consciousness during complex partial seizures with or without secondary generalization. Therefore, an ictal EEG (i.e. during the migraine attack) should be recommended in every migraine patients, even in subjects who are not known to be epileptic, although not always it is possible to detect an ictal epileptic manifestation only by scalp-EEG recording [27, 28].

## Discussion

Co-morbidity of migraine and epilepsy, although still under debate, is well known for more than a century [22, 32–35]. Patients with epilepsy and migraine are often cared for by different subspecialists (neurologists with subspecialty of migraine or epilepsy, paediatricians, general physicians, clinicians specialized in internal medicine, etc.), and, unfortunately, there is generally not enough communication among the different sub-specialists.

Although the terms migralepsy, hemicrania epileptica and post-ictal headache have been included in the recent ICHD-II classification, to date, neither the International Headache Society (HIS) nor the International League against Epilepsy (ILAE) mention that headache/migraine may be the sole ictal epileptic manifestation.

Ictal headache as the only clinical feature of an epileptiform EEG abnormality is very rare, and seizures associated with “migraine-like” manifestations probably represent an epileptic event rather than episodes with both migraine and epileptic mechanisms. In any case, when patients show features of both migraine and epilepsy, ictal EEG recordings should be performed to demonstrate the underlying pathogenic mechanism of these episodes; in some cases a PPR plays an important role [23]. Nevertheless, it should be taken into consideration that in some rare cases [27] only a depth electrode recording would be able to demonstrate an IEH [27], as it can also happen in other types of epileptic manifestations [28].

Literature data suggest that, even if clinical characteristics might be misleading [2, 3, 10], EEG and MRI findings allow correct diagnosis and treatment in most of these patients [4–7, 16–20]. Interestingly, diffusion-weighted MRI identified signal alterations in the region of seizure activity, confirming its utility to disclose the epileptic nature of status migrainosus/epilepticus [19, 20].

We are aware that the current ICHD-II criteria for migralepsy and HE have been made before publication of nearly all of the above reported cases [4, 18–20]. In any case, these five well-documented cases suggest that the term IEH could be a correct term to explain that headache can be the sole ictal epileptic manifestation; probably, most of the previously described cases of “migraine-epilepsy” (or migralepsy) simply started with an IEH followed by different associated sensory-motor manifestations.

## Conclusions and remarks

Based on the current knowledge and clinical experiences, migralepsy (coded in ICHD-II as 1.5.5 “migraine-triggered seizure”), is highly unlikely to exist as such. We, therefore, propose to delete this term until clear evidence is provided of its existence [16].

Ictal epileptic headache should be used to classify the events in which headache represents the only ictal epileptic feature [4, 17–20]; these rare cases should be classified as “autonomic epilepsy” [4, 16, 23, 36].

The term “Hemicrania epileptica” should be maintained in the classifications for all cases in which an IEH “co-exist” and is associated synchronously or sequentially with other ictal sensory-motor events.

## References

[1] Rogawski M (2008). Common pathophysiologic mechanisms in migraine and epilepsy. Arch Neurol.

[2] Panayiotopoulos CP (1999). Visual phenomena and headache in occipital epilepsy: a review, a systematic study and differentiation from migraine. Epileptic Disord.

[3] Panayiotopoulos CP (1987) Difficulties in differentiating migraine and epilepsy based on clinical EEG findings. In: Andermann F, Lugaresi E (eds) Migraine and Epilepsy, Butterworth, Boston, pp 31–46

[4] Parisi P, Kasteleijn-Nolst Trenite DGA, Piccioli M (2007). A case with atypical childhood occipital epilepsy “Gastaut type”: an ictal migraine manifestation with a good response to intravenous diazepam. Epilepsia.

[5] Parisi P (2009). Who’s still afraid of the link between headache and epilepsy? Some reactions to and reflections on the article by Marte Helene Bjørk and co-workers. J Headache Pain.

[6] Parisi P (2009). Why is migraine rarely, and not usually, the sole ictal epileptic manifestation?. Seizure.

[7] Piccioli M, Parisi P, Tisei P, Villa MP, Buttinelli C, Kasteleijn-Nolst Trenité DG (2009). Ictal headache and visual sensitivity. Cephalalgia.

[8] International Headache Society (2004). The International Classification of Headache Disorders: 2nd edition. Cephalalgia.

[9] Terzano MG, Pietrini V, Parrino L, Milone FF (1986). Migraine and intercalated seizures with occipital EEG paroxysms: observations on a family. Headache.

[10] Sances G, Guaschino E, Perucca P, Allena M, Ghiotto N, Manni R (2009). Migralepsy: a call for a revision of the definition. Epilepsia.

[11] Isler H, Wieser HG, Egli M, Andermann F, Lugaresi E (1987). Hemicrania epileptica: synchronous ipsilateral ictal headache with migraine features. Migraine and epilepsy.

[12] Lennox WG, Lennox MA (1960) Epilepsy and related disorders. Little, Brown, Boston

[13] Marks DA, Ehrenberg BL (1993). Migraine-related seizures in adults with epilepsy, with EEG correlation. Neurology.

[14] Berg AT, Berkovic SF, Brodie MJ (2010). Revised terminology and concepts for organization of seizures and epilepsies: report of the ILAE Commission on Classification and Terminology, 2005–2009. Epilepsia.

[15] Maggioni F, Mampreso E, Ruffatti S, Viaro F, Lunardelli V, Zanchin G (2008). Migralepsy: is the current definition too narrow?. Headache.

[16] Parisi P, Kasteleijn-Nolst Trenitè DGA (2010). “Migralepsy”: a call for revision of the definition. Epilepsia.

[17] Walker MC, Smith SJ, Sisodya SM, Shorvon SD (1995). Case of simple partial status epilepticus in occipital lobe epilepsy misdiagnosed as migraine: clinical, electrophysiological, and magnetic resonance imaging characteristics. Epilepsia.

[18] Ghofrani M, Mahvelati F, Tonekaboni H (2006). Headache as a sole manifestation in nonconvulsive status epilepticus. J Child Neurol.

[19] Perucca P, Terzaghi M, Manni R (2010). Status epilepticus migrainosus: clinical, electrophysiologic, and imaging characteristics. Neurology.

[20] Belcastro V, Striano P, Pierguidi L, Calabresi P, Tambasco N (2011). Ictal Epileptic Headache Mimicking Status Migrainosus: EEG and DWI-MRI Findings. Headache.

[21] Schön F, Blau JN (1987). Post-epileptic headache and migraine. J Neurol Neurosurg Psychiatry.

[22] Ekstein D, Schachter SC (2010). Postictal headache. Epilepsy Behav.

[23] Kasteleijn-Nolst Trenité DGA, Verrotti A, Di Fonzo A, Cantonetti L, Bruschi R, Chiarelli F, Villa MP, Parisi P (2010). Headache, epilepsy and photosensitivity: how are they connected?. J Headache Pain.

[24] Lauritzen M (1994). Pathophysiology of the migraine aura. The spreading depression theory. Brain.

[25] Calabresi P, Galletti F, Rossi C, Sarchielli P, Cupini LM (2007). Antiepileptic drugs in migraine: from clinical aspects to cellular mechanisms. TIPS.

[26] Andermann F, Zifkin B (1998). The benign occipital epilepsies of childhood: an overview of the idiopathic syndromes and of the relationship to migraine. Epilepsia.

[27] Laplante P, Saint-Hilaire JM, Bouvier G (1983). Headache as an epileptic manifestation. Neurology.

[28] Derry CP, Harvey AS, Walker MC, Duncan JS, Berkovic SF (2009). NREM arousal parasomnias and their distinction from nocturnal frontal lobe epilepsy: a video EEG analysis. Sleep.

[29] De Romanis F, Buzzi MG, Cerbo R, Feliciani M, Assenza S, Agnoli A (1991). Migraine and epilepsy with infantile onset and electroencephalographic findings of occipital spike-wave complexes. Headache.

[30] De Romanis F, Buzzi MG, Assenza S, Brusa L, Cerbo R (1993). Basilar migraine with electroencephalographic findings of occipital spike-wave complexes: a long-term study in seven children. Cephalalgia.

[31] Beaumanoir A, Andermann F, Beaumanoir A, Mira L, Roger J, Tassinari CA (1993). An EEG contribution to the study of migraine and of the association between migraine and epilepsy in childhood. Occipital seizures and epilepsy in children.

[32] Toldo I, Perissinotto E, Menegazzo F (2010). Comorbidity between headache and epilepsy in a pediatric headache center. J Headache Pain.

[33] Fernández-de-Las-Peñas C, Hernández-Barrera V, Carrasco-Garrido P, et al (2010) Population-based study of migraine in Spanish adults: relation to socio-demographic factors, lifestyle and co-morbidity with other conditions. J Headache Pain 11:97–104 10.1007/s10194-009-0176-5 PMC3452289 20012124

[34] Lipton RB, Ottman R, Ehrenberg BL, Hauser WA (1994). Comorbidity of migraine: the connection between migraine and epilepsy. Neurology.

[35] Ottman R, Lipton RB (1994). Comorbidity of migraine and epilepsy. Neurology.

[36] Ferrie CD, Caraballo R, Covanis A, Demirbilek V, Dervent A, Fejerman N, Fusco L, Grunewald RA, Kanazawa O, Koutroumanidis M, Lada C, Livingston JH, Nicotra A, Oguni H, Martinovic Z, Nordli DR, Parisi P, Scott RC, Specchio N, Verrotti A, Vigevano F, Walker MC, Watanabe K, Yoshinaga H, Panayiotopoulos CP (2007). Autonomic status epilepticus in Panayiotopoulos syndrome and other childhood and adult epilepsies: a consensus view. Epilepsia.

